# Morphological and Chemical Investigation of Ovarian Structures in a Bovine Model by Contrast-Enhanced X-ray Imaging and Microscopy

**DOI:** 10.3390/ijms24043545

**Published:** 2023-02-10

**Authors:** Alessandra Gianoncelli, Gabriela Sena Souza, George Kourousias, Ernesto Pascotto, Paul Tafforeau, Elena Longo, Regina Cely Barroso, Murielle Salomé, Marco Stebel, Federica Zingaro, Carla Calligaro, Giuseppe Ricci, Lorella Pascolo

**Affiliations:** 1 Elettra—Sincrotrone Trieste, Strada Statale 14—km 163,500 in AREA Science Park, Basovizza, 34149 Trieste, Italy; 2 Nuclear Engineering Department, Federal University of Rio de Janeiro, Rio de Janeiro 21941-972, Brazil; 3 ULSS n.2 Marca Trevigiana, Dipartimento di Prevenzione, Servizi Veterinari di Asolo, 31100 Treviso, Italy; 4 European Synchrotron Radiation Facility, 38043 Grenoble, France; 5 Physics Institute, State University of Rio de Janeiro, Rio de Janeiro 20550-013, Brazil; 6 Dipartimento Scienze Vita, Università di Trieste, V. Giorgieri 1, 34127 Trieste, Italy; 7 Physics Department, University of Trieste, Via A. Valerio 2, 34127 Trieste, Italy; 8 Servizio Diagnostica Veterinaria, University of Udine, 33100 Udine, Italy; 9 Institute for Maternal and Child Health, IRCCS Burlo Garofolo, 34137 Trieste, Italy; 10 Department of Medical, Surgical and Health Sciences, University of Trieste, 34149 Trieste, Italy

**Keywords:** ovary tissue, CT, microCT, µCT, XRF, contrast agents

## Abstract

An improved understanding of an ovary’s structures is highly desirable to support advances in folliculogenesis knowledge and reproductive medicine, with particular attention to fertility preservation options for prepubertal girls with malignant tumors. Although currently the golden standard for structural analysis is provided by combining histological sections, staining, and visible 2D microscopic inspection, synchrotron radiation phase-contrast microtomography is becoming a new challenge for three-dimensional studies at micrometric resolution. To this aim, the proper use of contrast agents can improve the visualization of internal structures in ovary tissues, which normally present a low radiopacity. In this study, we report a comparison of four staining protocols, based on iodine or tungsten containing agents, applied to bovine ovarian tissues fixed in Bouin’s solution. The microtomography (microCT) analyses at two synchrotron facilities under different set-ups were performed at different energies in order to maximize the image contrast. While tungsten-based agents allow large structures to be well identified, Iodine ones better highlight smaller features, especially when acquired above the K-edge energy of the specific metal. Further scans performed at lower energy where the setup was optimized for overall quality and sensitivity from phase-contrast still provided highly resolved visualization of follicular and intrafollicular structures at different maturation stages, independent of the staining protocol. The analyses were complemented by X-ray Fluorescence mapping on 2D sections, showing that the tungsten-based agent has a higher penetration in this type of tissues.

## 1. Introduction

The unique characteristics of synchrotron radiation (SR) allow for the development of advanced X-ray based techniques, which are becoming an exceptional tool in life science research, along with stimulating opportunities in biomedical imaging. Synchrotron facilities also offer the possibility of combining multiple approaches thus greatly enhancing the exploration of dynamical biological process occurring across various spatial resolution [1,2,3,4], from whole-body physiology down to the detection of single chemical species within individual cells.

Microtomography is one of the most attractive synchrotron techniques for the medical community, allowing the three-dimensional reconstruction of entire organs and portions of them [5], visualizing the sections with resolutions up to single-cell level and below [6,7,8]. The technique represents a unique challenge for basic and applied studies in reproductive medicine, allowing the investigation of reproductive organs as well as gamete differentiation and quality, both in normal and diseased conditions, particularly when applying synchrotron radiation.

The use of microCT (µCT) in in-vitro studies of the ovary is rather recent [9,10], and our group has already demonstrated the feasibility and utility of microCT experiments on human ovarian tissues [11]. Obviously, the development of an effective protocol to study these samples is fundamental for visualizing the features of interest as best as possible and to take full advantage of this powerful three-dimensional imaging mode.

The high coherence provided by most of the third and fourth generation synchrotron facilities allows the use of in-line propagation phase-contrast microtomography, a technique that helps to enhance the contrast in low-density tissues [12]. However, for samples which present similar densities among different tissues or structures, simple in-line phase-contrast technique may not be able to provide images with enough contrast to distinguish the different structures. In this situation, the use of a correct staining protocol is the solution and can dramatically improve the image quality [13]. Several protocols have been developed and investigated in recent years on different types of tissues, highlighting advantages and disadvantages of different staining procedures. Metscher compared simple staining methods using a commercial microCT system to study embryonic tissues and insects and demonstrated that iodine, phosphotungstic acid (PTA), and osmium can be excellent staining if used correctly [14]. PTA is simple and produces excellent contrast, but its penetration in the tissues is much slower than for iodine; therefore, the samples usually need to stay longer in these solutions [9]. Metscher also demonstrated that osmium provides excellent results in relation to the contrast between the tissues; however, iodine and PTA are easier to handle and much less toxic. From a translational point of view, and considering the perspective of in-vivo applicability, Xia et al. [10] showed how Lugol’s Iodine (I2-IK)-enhanced microCT is becoming an emerging tool to image tumor specimens both in animal and human beings, accurately demonstrating the position of the tumor. µCT can also be performed on samples simply infused with ethanol since it acts as a negative contrast agent that can also penetrate very fast on large samples, is inexpensive, and ensures stability over a long time with limited danger [5].

In this work, four different staining protocols were applied in order to specifically study in-vitro ovary tissue morphology using synchrotron radiation propagation phase-contrast microtomography (SR-µCT). For obvious ethical reasons, the study was performed on bovine tissues, but the results can be extended to human ones if this precious material could be accessed. A proper procedure to investigate in 3D, for instance, the quality and state of cryopreserved human ovary tissues would have important consent in the medical community dealing with fertility preservation [11,15,16,17].

When setting cryopreservation protocols for ovary tissues, the contained follicles present are evaluated for quality by visible light and/or electron microscopy, [18,19,20] after slicing the tissues to thicknesses suitable for histological analyses [21,22,23]. A three-dimensional visualization of these structures directly inside the tissues could provide new information about their development/growth. The use of microtomography in studies of the ovary is very recent [11,24], and our group has already demonstrated the feasibility and utility of microCT experiments on human ovarian tissues [11]. The development of an effective protocol to study these samples is fundamental for visualizing the features of interest as best as possible and for taking full advantage of this powerful three-dimensional imaging mode.

The main advantages of the chosen staining presented in this work, Lugol’s solution, PTA, and iodine solutions at different incubation times, are the simplicity of the sample preparation and their safer nature compared to others, such as osmium [25].

Iodine staining, with iodine solution or Lugol’s Solution, is the most widely used among the contrast agents and is known for its fast tissue penetration, cost effectiveness, and affinities with different biological tissues [26]. In particular, small specimens can be stained with low concentrations (i.e., ≤1% *w*/*v*) of Lugol’s solution or iodine solution, because iodine needs shorter distances to reach internal soft tissues [27].

In addition to iodine, another widely used staining in biological and medical samples is PTA, which produces excellent tissue contrast and is simple to handle [28].

In this work, the bovine ovary samples, treated with the above mentioned four protocols, were analyzed at two microCT beamlines, ID19 [29,30] at ESRF synchrotron (Grenoble, France) and at SYRMEP beamline [31] at Elettra Sincrotrone Trieste (Trieste, Italy), using different spatial resolutions. The first scans were taken at the ID19 beamline with a pixel size of 2.2 μm. The regions containing follicles were identified based on these first tomography results, and some regions were re-scanned focusing on a smaller field of view and using a lower pixel size (0.9 μm) at SYRMEP.

The results obtained in this research have shown significant interesting differences between samples measured with different protocols. The PTA protocol provided the highest contrast although all protocols were effective under synchrotron radiation.

Results were combined with X-ray Fluorescence (XRF) measurements at two incident energies (1.5 and 7.3 keV) on histological slices obtained from the analyzed samples: the data further support the benefit of using PTA, presenting an increased contrast efficiency because of its higher penetrance in tissues.

The microtomography approach could offer a unique opportunity for resolving ovary structures or for evaluating the quality of ovarian fragments before and after cryopreservation, the latter in the context of autotransplantation protocols [32] of ovarian tissues for adolescent oncological patients. A better knowledge of the three-dimensional structure of human ovaries could also favor the setting of the artificial ovary, which is the most challenging and risky perspective in reproductive medicine [33,34,35,36,37].

## 2. Results

### 2.1. Microtomography

Bovine ovary tissues stained with the above-mentioned staining protocols were scanned by X-ray microtomography. From the single distance set of projections, a three-dimensional view of the tissues was reconstructed, allowing the inspection of the tissues from different views and the isolation of objects.

**Figure 1:**
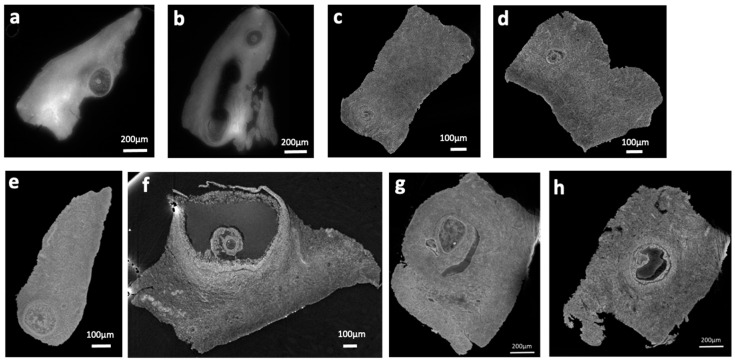
(**a**–**h**) Slices reconstructed from the microCT scans: PTA stained (**a**,**b**); Iodine stained for 2 h (**c**,**d**); Lugol’s stained (**e**,**f**); Iodine stained overnight (**g**,**h**). See the text for energy. CT scans were performed at ID19 with a pixel size of 2.2 μm.

Figure 1 shows representative longitudinal sections from the ovary samples stained with the four protocols. All the protocols used for microCT studies were successful in providing a precise visualization of antral and preantral follicles, corpus luteum, and, more importantly, some follicles at early stages of development.

Panels a and b in Figure 1, obtained from two samples, demonstrate the high penetration of PTA staining in the tissue after the 2 h of incubation although, in one of them (Figure 1b), the most internal part of the specimen has not been totally reached.

Panels c, d, g, and h show slices of samples stained with Iodine solution for 2 or 24 h. The staining appears efficient in revealing macroscopic tissue details, and no substantial contrast difference can be appreciated from the comparison of the two incubation times. As for iodine staining, Lugol’s (Figure 1e,f) staining uniformly penetrated the tissue but seems to provide a smaller contrast. As expected, the PTA provides a better visibility of large structures, while iodine makes the fine structures clearer. It is linked to the very different Z of the W and I elements. As we used single distance phase retrieval, the delta/beta (ratio between phase and absorption) difference is much stronger between organic material and PTA than between organic material and iodine. As a result, a single distance phase retrieval process as we used will tend to make the effect of the contrast agent blurrier in the case of higher Z. It would tend to obscure a bit the very fine structures.

In line with this, by selecting and examining at least three ROIs (Region Of Interest) from a few regions containing follicles, the contrast-to-noise ratio (CNR) was calculated using the formula described by Liu et. al. [38]. Regions within the follicles and external ones were selected for CNR calculation. All the selected ROIs have the same area. The CNR provides quantitative information of an image about the contrast enhancement and the noise level [39]. The values obtained from this parameter resulted in 1.8, 3.9, and 7.3 for Lugol’s solution, iodine solution, and PTA-stained tissues, respectively, implying that PTA staining provides a better contrast and visibility for large structures compared with the other staining solutions, in the conditions used in the present experiments.

**Figure 2:**
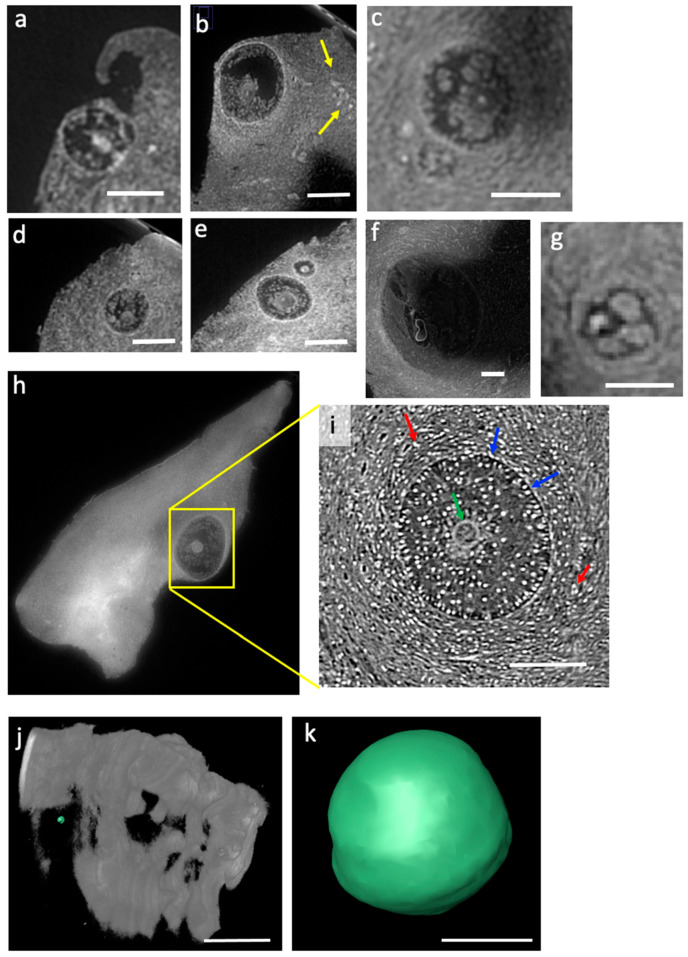
(**a**–**g**) Reconstructed slices from the microCT scans on the PTA-stained tissue at 69.5 keV at ID19 beamline with 2.2 μm pixel size. The scale bar is 100 μm. (**h**) Slice extracted from the whole PTA-stained sample. (**i**) corresponding follicle region measured at 22 keV at SYRMEP beamline with 0.9 μm pixel size; the scale bar is 60 μm. (**k**) Volume of a follicle virtually extracted of PTA-stained tissue (calculated volume: 8565 μm^3^); (**j**) Segmentation representing the area where the follicle was extracted. The scale bar in (**j**) is 250 μm. The scale bar in (**k**) is 15 μm.

The tomographic reconstruction of Video S1 (Supplementary Materials) allows easy identification of several follicles at different maturation stages in the ovary cortex. Small vascular structures can be inferred in Figure 2b (see yellow arrows). Some follicles are better highlighted in Figure 2 panels a–f where the follicle region presents highly contrasted structures: the theca cells around the follicle are particularly evident as well as the oocyte inside the follicle. The remaining part of the follicle appears instead less contrasted, allowing its quick identification while browsing the video. In some regions, some physiologically damaged follicles could also be detected (Figure 2g), demonstrating the high sensitivity provided by the applied experimental conditions. To note, the images were acquired at 69.5 keV energy, just above W K-edge, in order to maximize the contrast of the staining. Prior to that, we performed scans at three different energies (35 keV, 64 keV, and 69.5 keV) on the same PTA sample to determine the best experimental condition for maximal contrast in the images. Similar tests were performed across the Iodine K-edge.

A sub-region of one of the PTA-stained tissues was also analyzed at SYRMEP beamline at lower energy and higher spatial resolution. Hence, in Figure 2h,i, we compare the same follicle imaged in the two experimental conditions. Even if the follicle shown in Figure 2i was not acquired at the highest contrast condition, as in Figure 2h, the phase contrast modality deployed in SYRMEP was efficient in revealing micrometric details that precisely delineate the oocyte membrane (green arrow), and the theca (blue arrow) and stroma cells (red arrows). Despite the fixation procedure, the follicle appears intact and well defined. Although the energy used at SYRMEP was below W K-edge, it allows for a good visualization of staining agents because there is a higher difference between the attenuation of tissue and that of the contrast agent itself. Indeed, in the ID19 images above the W K-edge, the oocyte appears much brighter than the rest of the follicle, while on the SYRMEP ones, whose configuration was much more adapted to see the fine structures but less sensitive to the overall staining effect, the oocyte is at the same grey level as the rest of the follicle: only the fine structures are apparent, coming from the propagation effect at high resolution that is not sensitive to the low frequencies.

The resolution and contrast obtained at SYRMEP also allows the monitoring of single follicle status and size, as visible in Figure 2k where a representative gamete has been virtually extracted (see Figure 2j), allowing the determination of its volume.

The follicle was semi-automatically segmented using the Avizo 8.0 software, and the volume was calculated through the Volume Edit tool in this software. All the segmentations and volume calculations in this paper were performed using the same procedure.

**Figure 3:**
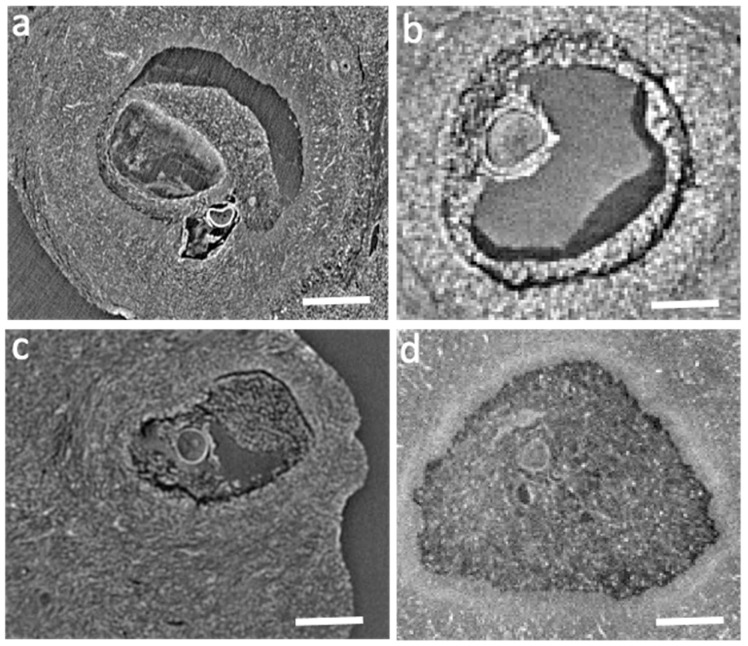
(**a**–**d**) Slices reconstructed from the microCT scans (at 33 keV at ID19 beamline, with 2.2 μm pixel size), iodine stained (2 h incubation). The scale bar is 100 μm.

Panels a,b,c,d in Figure 3 show the reconstructed slices sequence from the tomographic reconstruction of Video S2 (Supplementary Materials) of an ovary sample stained with the iodine solution protocol. Even if all tissue structures seem to show a similar radiopacity, this protocol is effective in revealing antral and preantral follicles and their internal texture. The oocytes are well revealed too. Sometimes the follicles appear detached from the stromal tissue, possibly as a result of the fixation procedures.

**Figure 4:**
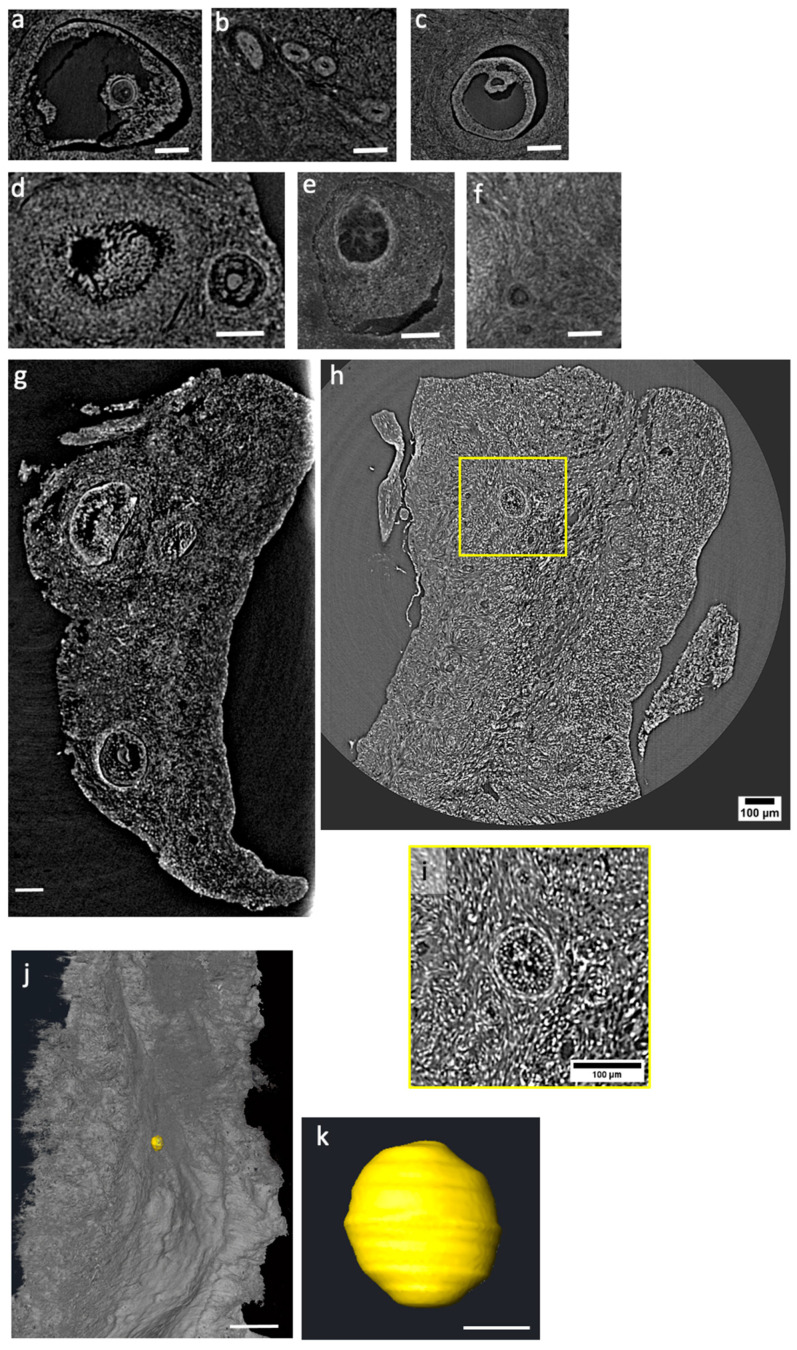
(**a**–**j**) Slices reconstructed from the microCT scans (at 33 keV at ID19 beamline with 2.2 μm pixel size), iodine-stained overnight incubation. Scale bars are 100 μm. (**g**) Slice extracted from the whole iodine-stained overnight sample. (**h**) Slice extracted from the whole iodine-stained overnight sample at 27 keV at SYRMEP beamline with 0.9 μm spatial resolution with a zoom (**i**) on a preantral follicle. (**k**) Volume of an oocyte virtually extracted from slice (**j**) (calculated volume: 9362 μm^3^); The scale bar in (**j**) is 100 μm. The scale bar in (**k**) is 30 μm.

Similar results were obtained with the iodine protocol with 24 h incubation, as shown in Video S3 (Supplementary Materials) and in Figure 4. The video confirms a comparable, quite uniform penetration of the staining across the whole specimen. Slices extracted from the tomographic reconstruction of Video S3 are shown in Panels a–f in Figure 4. Similarly, to the protocol with 2 h incubation, there is an efficient visualization of the different tissue structures; oocytes (or primordial follicles) with dimension down to 20 microns in diameter can be clearly identified as demonstrated from Panel j in Figure 4.

**Figure 5:**
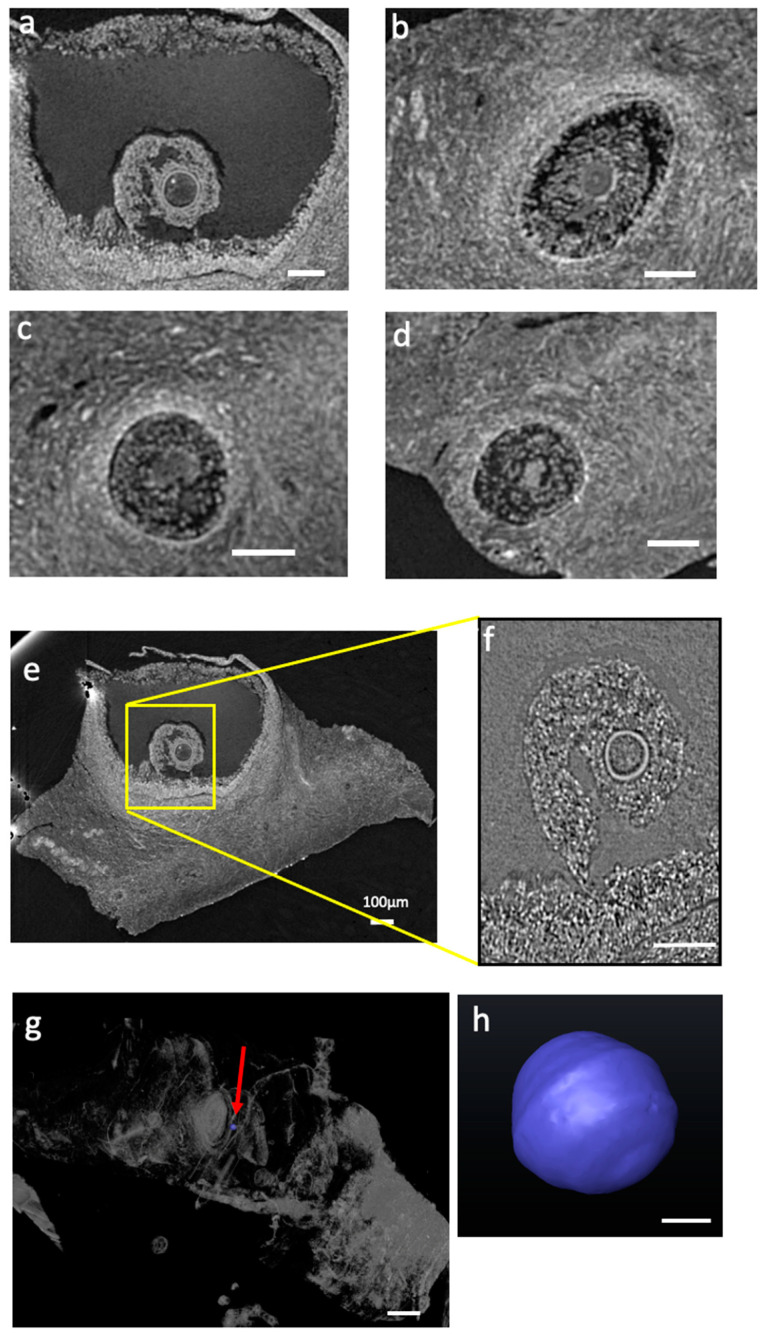
(**a**–**e**) Slices extracted from the microtomography reconstruction (at 33 keV at ID19 beamline with 2.2 μm pixel size), Lugol’s-stained (2 h incubation). The scale bar is 100 μm. (**e**) Slice extracted from the whole Lugol’s-stained sample and (**f**–**h**) corresponding follicle region measured at 22 keV at SYRMEP beamline with 0.9 μm pixel size. The scale bar is 250 μm (**f**). (**h**) Volume of an oocyte virtually extracted from the red arrow site indicated in the slice depicted in panel (**g**) (calculated volume: 33,204 μm^3^). The scale bar is 20 μm.

Panels a,b,c,d in Figure 5 show slices extracted from Video S4 (Supplementary Materials) from an ovary sample stained with the Lugol’s solution protocol. In Figure 1b, the follicle region can be shown, showing poor contrast inside the follicle. Compared to iodine staining, Lugol’s protocol seems very similar, perhaps better highlighting the theca cells, while providing smaller radiopacity on the stroma cells, in agreement with the calculated CNR value, demonstrated to be the lowest one. However, the analysis at SYRMEP on a selected antral follicle (Figure 5f, well reveals the histological details of the follicle and oocyte). The empty space around the follicle surely increases the differential signal. In this experimental condition, oocytes can also be visualized as shown in Figure 5h, extracted from the Lugol’s-stained tissue slice (see Figure 5g).

### 2.2. X-ray Microscopy and XRF Analyses

Ten-micron thick slices obtained from PTA and iodine-stained ovary tissues were analyzed by XRF microscopy in order to investigate the staining distribution and potential targeting of specific structures. Lugol’s-treated tissues were not investigated due to the low contrast demonstrated by micro-tomography, as shown in Figure 5. Low and mild energy XRF were combined to complement light and heavier element distribution.

**Figure 6:**
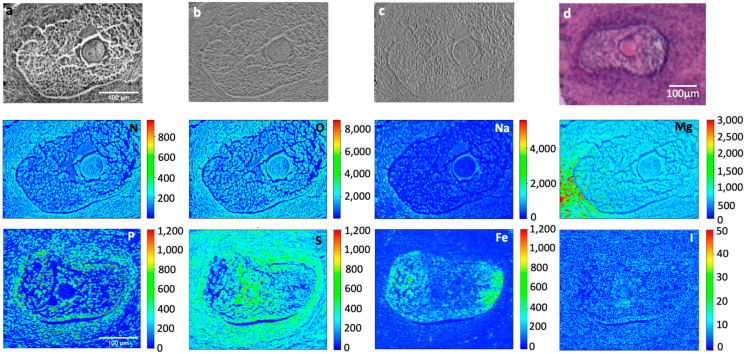
µXRF and X-ray microscopy of (24 h) iodine-stained bovine ovarian tissue. The absorption (**a**) and phase contrast (**b**,**c**) images were measured at the TwinMic beamline with 1.5 keV photon energy and 1 µm spatial resolution, together with the corresponding Na, Mg, N, O, and W XRF maps (350 µm × 250 µm) showing the distribution of different elements. The bottom panels show P, S, Fe, and W XRF maps (180 µm × 155 µm) acquired at ID21 beamline at 7.3 keV and 2 µm spatial resolution. (**d**) panel show the histological image of the oocytes from an adjacent slice stained with eosin and hematoxylin dye. Scale bars are 100 µm.

**Figure 7:**
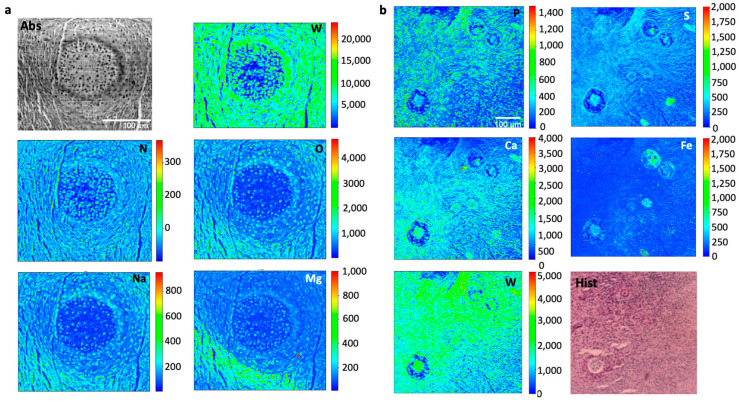
(**a**) µXRF and X-ray microscopy images of PTA-stained bovine ovarian tissue. The absorption (Abs) image was acquired at the TwinMic beamline with 1.5 keV photon energy and 1 µm spatial resolution, together the corresponding Na, Mg, N, and O XRF maps (300 µm × 220 µm), showing the distribution of the different elements, while the W XRF map was collected at 2 keV. (**b**) P, S, Ca, Fe, and W XRF maps (200 µm × 190 µm) acquired at ID21 beamline at 7.3 keV and 1 µm spatial resolution, collected on PTA-stained bovine ovarian tissue adjacent to the depicted histological one (Hist) stained with eosin and hematoxylin dye. Scale bars are 100 µm.

The soft X-ray microscopy absorption and phase contrast images (Figure 6a–c and Figure 7a) show that both iodine and PTA staining provide a good contrast which allows the identification of the different tissue structures, both for follicles and surrounding stromal tissue.

Figure 6 depicts a representative antral follicle from an iodine-stained tissue (24 h). Despite the fixative and staining treatments, most detectable light elements (N, O, Na, Mg, P, and S) maintain a characteristic distribution with a higher presence in the stromal cells compared to the intrafollicular fluid, allowing the oocyte to be well identified. Interestingly, there is a high presence of Fe inside the follicle which is compatible with its advanced maturation stage [40]. The iodine signal appears to be quite low: since iodine contrast is evident in microtomography, it implies that this staining is somehow washed out during sample preparation for XRF measurements. Indeed, after cutting the tissues, the 5 μm thick slices are collected from lukewarm water, which clearly affect iodine content, as this dye is water soluble.

Similar considerations apply for the PTA-treated tissue (Figure 7) where again Fe appears to be present mainly inside the follicles. Interestingly, the tissue shows a high Ca signal with a distribution delineating tissue’s and cell’s structure, somehow recalling that of phosphor. On the other hand, in the iodine-treated sample Ca distribution does not appear to match tissue morphology, and its signal is one order of magnitude lower. Interestingly, by inspecting W signal we can affirm that PTA staining is well absorbed by the whole tissue and appears high and almost uniformly distributed in the stromal area.

## 3. Discussion

This study describes synchrotron microCT analyses performed at two different synchrotron facilities on bovine ovarian tissue, using three different staining compounds already employed for biological specimens. It has been already demonstrated [14] that MicroCT imaging combined with suitable radiopaque staining can provide quantitative, high-resolution, high-contrast volume images of soft tissues, without excessive damage for the specimens, also in the field of reproductive medicine [11]. The staining protocols do not exclude the possibility of combining micro-CT with other preparation and analytical techniques. Indeed, phase contrast based micro-CT provides excellent soft tissue contrast, even on hard X-rays, as already highlighted by several successful biomedical applications [41,42,43,44,45], overcoming the limitations of traditional absorption contrast tomography.

In our previous study [11], we showed how synchrotron-based microCT can exploit phase contrast to successfully visualize with a high definition the anatomical structures inside ovarian tissue specimens, even early stage (primordial and primary) ovarian follicles and vascularity. Compared to that study where we used a fixation and staining typical of TEM microscopy, in the current one the specimens were fixed with Bouin’s solution following an already published protocol [46], prior to adding the dyes. As previously shown in the figures and videos, in our case the chosen fixation appeared to be adequate to maintain a good histology, even though in some cases the penetration of the staining was not uniform, as already found by other authors [47]. However, the CT images appeared to be well contrasted, likely due to a differential staining uptake from the different tissue structures. In agreement with this, the morphology showed by XRF microscopy imaging is well maintained, even if iodine presence seems to be greatly reduced compared to PTA, which is compatible with an almost total wash out during the histological sectioning. Interestingly, PTA does not suffer from the same issue, since it cross-links steadily with collagen and other macromolecules of the tissue [48].

In particular for the present study, the CT-imaging was performed at two synchrotron facilities, each exploiting its peculiar capabilities. At ID19 beamline of ESRF, the incident energy was chosen accordingly to the staining protocol, namely 33 keV for the iodine protocol and 69 keV for the PTA one, in order to take advantage of the absorption edge of iodine and tungsten, respectively. The 2.2 micron pixel size used at ID19 was the best compromise at the applied energies to preserve the integrity of the specimens and to allow a bigger field of view for scanning the whole sample. The ID19 experimental set up seems to favor PTA staining, allowing the easy identification of theca and granulosa cells and also follicles at early developmental stages, likely due to an increased penetration of the contrast agent in the samples. In particular, the tungsten-based agent allowed large structures in the tissue to be well identified, while iodine ones better spotted smaller features, especially by acquiring the images above the K-edge energy of the specific metal.

At SYRMEP beamline, the experimental conditions implied the use of a fixed energy well below the K-edge energy of both metals and higher resolution with a setup optimized for phase contrast imaging. The obtained reconstructions showed very good contrast with all staining, apparently not substantially influenced by the contrast agents, allowing internal structures such as follicular and intrafollicular structures at different maturation stages to be highlighted independently of the staining protocol and in a better way than what we obtained with osmium staining [11].

Finally, microCT preserves the condition of the specimen, enabling its further analysis with other analytical methods, such as X-ray Fluorescence microscopy.

## 4. Materials and Methods

### 4.1. Sample Preparation

Ovarian tissue samples were obtained from 6 young heifers at a slaughterhouse (macello Pelizzari, Loria, Treviso, Italy). All samples were collected into ice-cold PBS and fixed Bouin’s Fluid for 3 h and then maintained in 70% alcohol until the staining.

For the staining, some samples were maintained for 24 h in a Lugol’s Solution (I2KI), composed of one part of elemental iodine with two parts of potassium iodide [49,50]. Then they were dehydrated and included in paraffin.

The second set of samples was immersed in a 1% *w*/*v* PTA alcoholic solution (70%) (phosphotungstic acid) and kept for 2 h. Then the samples were included in paraffin [39].

The third set was immersed in a 1% *w*/*v* iodine alcoholic solution (70%) for 2 or 24 h and then included in paraffin.

For micro-tomography, the whole tissue included in the paraffin block was analyzed. For X-ray microscopy, the tissues were sectioned with a microtome in slices of 5 micron thickness; the cut slices were collected from lukewarm water and deposited onto 4 μm-thick ultralene foils (SPEX SamplePrep, purchased from Exacta+Optech Labcenter Spa, Modena, Italy). Consequent slices were stained with eosine and hematoxylin for inspecting cell nuclei and extracellular matrix in order to guide the X-ray microscopy analyses and identify the most significant regions to be mapped.

All chemicals, where not differently indicated, were purchased from Sigma Aldrich (Milano, Italy).

### 4.2. Microtomography

#### 4.2.1. Scans at ID19 Beamline (ESRF)

Phase-contrast microCT scans were performed in the propagation-based modality using the set-up available at the ID19 beamline of ESRF synchrotron (Grenoble, France).

A PCO edge 5.5 camera coupled using a tandem optic (Hasselblad 100 mm f/2.2/Hasselblad 300 mm f/4) mounted with a LuAG:Ce 100 µm thick scintillators was used to achieve a 2.2 μm pixel size. For the samples with iodine and Lugol, the beam from the U17.6 undulator set at a gap of 13 mm was filtered with 3.5 mm Al and 0.14 mm Cu, resulting in an average energy of 37 keV. It was selected to be above the K-edge of the iodine (33 keV) in order to maximize the absorption contrast. The samples with PTA were scanned with an average energy of 69.5 keV obtained by filtering the beam from the W150 wiggler set at a gap of 40 mm filtered with 0.28 mm gold and 2.8 mm of aluminum. Prior to selecting these experimental conditions, PTA samples were also scanned at 35 keV and 64 keV and compared with the results at 69.5 keV, to verify the optimal conditions for maximal contrast in the images. This setup gives a pink beam spectrum just above tungsten K-edge, enhancing the absorption from the contrast agent. The general scanning parameters were 3000 projections of 4 × 25ms each, and the propagation distance was 110 cm.

Tomographic slices were reconstructed using the conventional filtered back projection algorithm, and the approach to the inverse problem of phase retrieval based on the Paganin et al. [51] algorithm was used. Image processing of the 3D volume was carried out using Avizo 8.0 software.

#### 4.2.2. Scans at SYRMEP Beamline (Elettra)

Phase-contrast microCT scans were performed in the propagation-based modality using the set-up available at the SYRMEP beamline [31] of the Elettra synchrotron facility (Trieste, Italy).

A lens-coupled sCMOS camera system (Hamamatsu C11440-22C-Flash4.0 v2) equipped with optics designed to achieve up to 0.9 μm spatial resolution was used in white X-ray beam mode filtered by a 1 mm Si, with an average energy of 22 keV. The sample-to-detector distance was set to 15 cm in order to achieve the phase-contrast effects. For our experiments, 1800 radiographic projections were acquired over an angular range of 180°. The microtomographic slices are reconstructed from a series of 2D projection images acquired by performing a rotational scan over 180°, which is a very straightforward process compared to serial sectioning [52]. Tomographic slices were reconstructed using the conventional filtered back projection algorithm using the SYRMEP TOMO PROJECT (STP v.112022a) software developed by the SYRMEP team [53]. The approach to the inverse problem of phase retrieval based on transport-of-intensity (TIE) algorithm [51] was used. Image processing of the 3D volume was carried out using Avizo 8.0 software.

### 4.3. X-ray Fluorescence Microscopy

Slices of tissues stained with iodine and PTA protocol were analyzed by low-energy X-ray fluorescence (LEXRF) [54,55] combined with scanning transmission X-ray microscopy at TwinMic beamline [56,57] of Elettra Sincrotrone Trieste (Trieste, Italy). The incident X-ray beam energy was 1.5 keV to ensure the best excitation and detection of the Kα lines of Mg and Na atoms. The PTA sample was also measured at 2 keV to excite W M lines. The samples were raster-scanned with step size of 1µm across a microprobe of 1.2 µm delivered by a 600 µm diameter Au zone plate optics with 50 nm outermost zone.

Adjacent tissues slices were analyzed at ID21 beamline of ESRF synchrotron [58] where the samples were raster scanned at 2 µm micron step size and 7.3 keV incident beam in order to probe, among others, also the L lines of iodine and M lines of tungsten.

XRF spectra were then processed with PyMCA software (v.5.9.7) [59].

## Data Availability

The LEXRF and STXM raw and processed data and metadata are publicly available in a suitable repository in Elettra Sincrotrone Trieste for Open and FAIR scientific data [60]. All other data that support the findings of this study are available from the corresponding author upon request.

## Supplementary Materials

Supplementary materials can be found at https://doi.org/10.5281/zenodo.7459032 (accessed on 19 December 2022).

## Abbreviations

SR	Synchrotron Radiation
MicroCT, µCT	Microtomography
PTA	Phosphotungstic acid
XRF	X-ray Fluorescence
